# Selective Paste Intrusion—Effect of Process-Integrated Welding of Reinforcement Segments on Bond Strength

**DOI:** 10.3390/ma19214459

**Published:** 2026-10-23

**Authors:** Alexander Straßer, Thomas Kränkel, Christoph Gehlen

**Affiliations:** Chair of Materials Science and Testing, Centre for Building Materials (cbm), School of Engineering and Design, Technical University of Munich, 85748 Garching, Germany; thomas.kraenkel@tum.de (T.K.);

**Keywords:** selective paste intrusion, wire arc additive manufacturing (WAAM), additive manufacturing, bond behaviour, reinforcement integration, thermal exposure, welding, stud welding, push-through test, particle bed

## Abstract

Integrating Wire Arc Additive Manufacturing (WAAM) into the Selective Paste Intrusion (SPI) process enables the fully additive fabrication of reinforced concrete structures with complex geometries. However, the WAAM process transfers thermal energy into the reinforcement and the surrounding concrete matrix, which may affect the bond between steel and concrete. This study addresses whether the heat introduced by the welding of reinforcement segments impairs the bond between reinforcing steel and SPI-fabricated concrete. To isolate this thermal effect from the geometric process effects covered in a companion study, two groups of 16 mm reinforcing bars embedded in SPI-fabricated concrete were compared: bars whose segments were joined during printing by manual welding at seven joints per bar (process-integrated thermal exposure), and bars with adhesively bonded segments without heat input as the reference. Eight specimens per group from two production series were tested in push-through. A grey-body estimate based on the thermography and an assumed steel emissivity of 0.20 to 0.30 indicates approximate bar-surface temperatures of at least 214 to 277 °C near the joints immediately after welding and of 125 to 162 °C after 2 to 3 min. Despite these temperatures and the repeated heating and cooling cycles during printing, no discernible difference in bond capacity from the adhesively bonded reference group was detected: the welded group reaches a mean maximum bond stress of 20.5 MPa, the adhesively bonded reference 22.4 MPa, and the difference of 1.9 MPa (9%) lies within the scatter of the two groups (coefficients of variation 22% and 21%). With a 95% confidence interval of −7.0 to 3.1 MPa for this difference, a reduction of up to 31% cannot be excluded with this sample. All specimens of both groups failed by bar push-through, with a similar slip at maximum bond stress. Because the weld beads also change the joint geometry, the thermal exposure cannot be fully separated from the joint geometry. Since the procedure investigated here resembles a stud welding process rather than genuine WAAM fabrication, the results indicate that joining reinforcement segments by stud-welding-like processes during printing is a feasible route for integrating reinforcement into SPI with respect to the bond under short-term compressive loading. For genuine WAAM, further investigations are required, since welding occurs far more frequently than in the present procedure because of the smaller layer heights, so that heat accumulates and overlapping thermal cycles can produce even higher sustained temperatures at the steel–concrete interface.

## 1. Introduction

**Figure 1:**
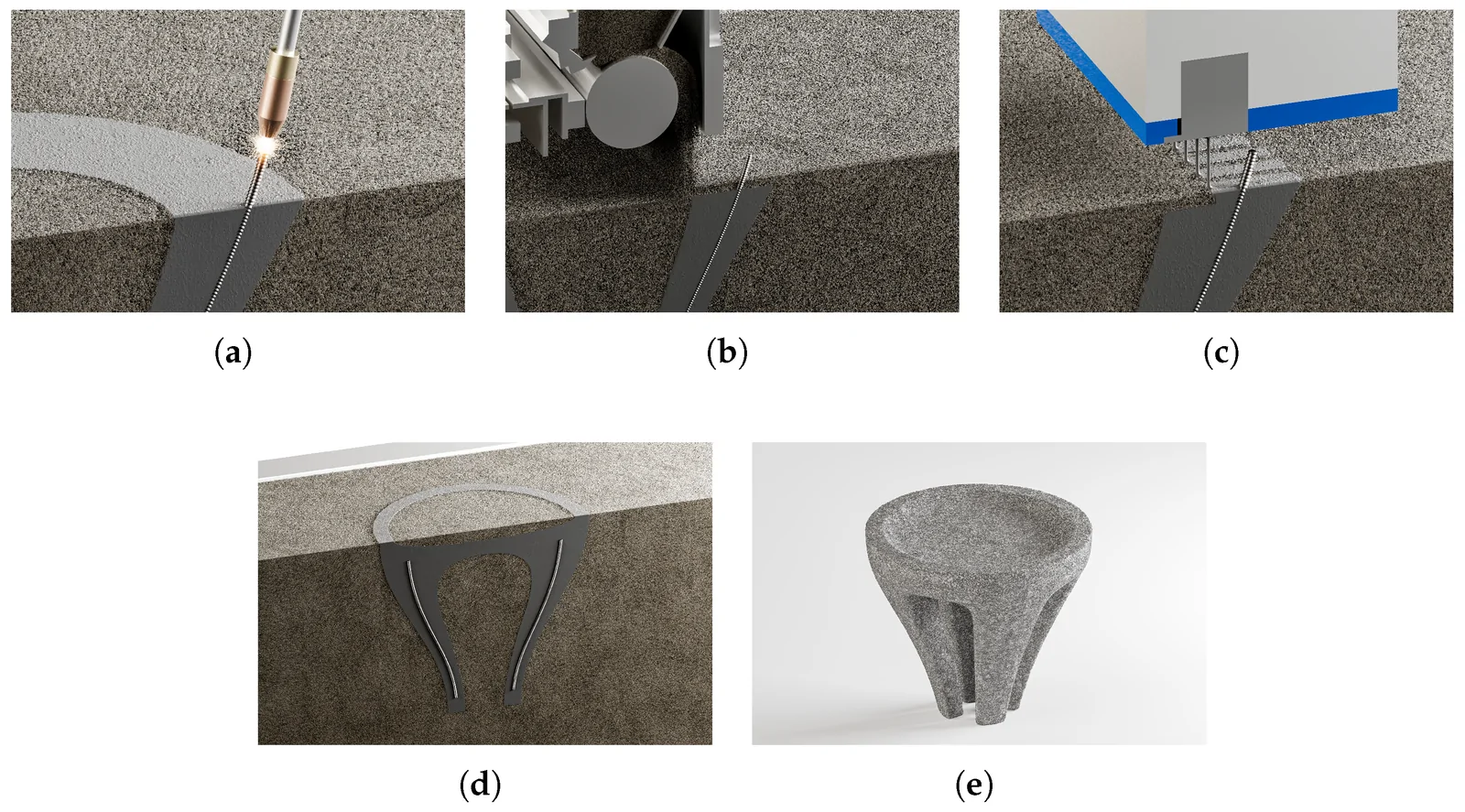
Schematic representation of the combined SPI and WAAM process (renderings), modified after [3]. (**a**) Printing a reinforcing bar. (**b**) Spreading an aggregate layer. (**c**) Applying cement paste. (**d**) Curing the finished reinforced concrete element. (**e**) Excavating the component.

Selective Paste Intrusion (SPI) is an additive manufacturing method for producing concrete elements with complex geometries. Aggregates are sequentially spread to form a particle bed, and cement paste is selectively applied to bind them, while the unbound surrounding aggregates act as temporary support, so that freeform components can be fabricated without formwork (Figure 1) [1,2].

Strategies for integrating reinforcement into digitally fabricated concrete are the subject of ongoing research [4,5]. Combining SPI with Wire Arc Additive Manufacturing (WAAM) has been proposed as a strategy to produce custom-shaped reinforcement directly within the particle bed [6]. WAAM reinforcement reached upper yield strengths of 316 to 348 MPa, tensile strengths of 454 to 481 MPa, and uniform elongations of 14.4 to 17.5% in the valid tensile tests, and its bond performance is comparable to conventional B500B reinforcement [7]. B500B denotes a characteristic yield strength $f_{\mathrm{yk}}$ of 500 MPa and ductility class B according to DIN EN 1992-1-1 [8], the latter defined by a strain-hardening ratio $k={\left(f_{t}/f_{y}\right)}_{k}\geq 1.08$ and a characteristic strain at maximum force of at least 5.0%. By yield strength, B500B lies between the specified minimum yield strengths of ASTM A615/A615M Grade 60 (420 MPa) and Grade 80 (550 MPa) [9]. The combined process alternates between steel deposition by WAAM, aggregate spreading, and selective paste intrusion, building the reinforced concrete element layer by layer inside the particle bed. A related hybrid concept has been proposed for extrusion-based printing: in the Additive Manufacturing of Reinforced Concrete (AMoRC) process, segmented reinforcing bars are joined by intermittent stud welding ahead of a continuous concrete extrusion [10,11]. The combination of in situ arc welding with a particle-bed process, in which the selectively intruded fresh matrix surrounds the reinforcement during welding, has to the authors’ knowledge only been investigated for SPI.

Bond between ribbed reinforcement and concrete is transferred by adhesion, mechanical interlock of the ribs, and friction, which dominate successively with increasing slip [12,13,14,15]. Damage to the matrix around the bar, whether thermal or from impaired material deposition, can therefore affect both the early bond response and the bond capacity.

A challenge of this combination is the heat generated during the WAAM process. In WAAM, every deposited layer reheats the material below it, and with short interlayer times the heat accumulates over successive layers, which raises the interlayer temperature and changes the microstructure and mechanical properties of the deposit [16]. Interlayer dwell times and active or forced interpass cooling are therefore used to control this thermal history [17,18]. In a combined SPI-WAAM process, the accumulated heat would also act on the fresh cement paste and on the forming steel–concrete interface. The thermal load transferred from the welding point into the reinforcement and the surrounding particle bed can alter the rheological properties of the cement paste and impair the mechanical performance of the hardened concrete. Cement-paste rheology is sensitive to temperature, with changes in yield stress and plastic viscosity reported within 20 to 50 °C [19,20,21].

For the SPI system considered here, the relevant thermal limits were established in a sequence of preceding studies. The fresh-state limit was identified by measuring how rising cement paste temperatures affect yield stress and viscosity. A sharp transition was observed at 60 to 70 °C, above which penetration of the cement paste into the particle bed deteriorates [22]. The hardened-state limit was established by examining the effect of fresh-state thermal exposure on the compressive and flexural strength of the resulting concrete, which decline at a fresh-state temperature of 80 °C and above [23]. The actual temperatures occurring during WAAM at varying distances from the welding point, and corresponding cooling strategies, were then quantified [24]. Depending on the distance to the welding point, the reinforcement reached temperatures of 100 to 450 °C at an interlayer cooling time of 60 s [24]. Bar temperatures of 164 to 206 °C were measured by thermocouples embedded in the bar at a distance of 40 mm between measuring point and process zone [24]. These temperatures exceed the identified rheological and hardened-state thresholds.

To reduce the thermal load on the particle bed, one strategy is to increase the vertical distance between the welding point and the bed surface. Print quality is unimpaired up to 40 mm, and a first strength reduction of about 16% sets in at 50 mm [3]. This vertical protrusion of the reinforcement gives rise to shadowing effects, a process-induced disturbance of material deposition in the vicinity of the protruding bar. A companion study [25] investigates these geometric effects systematically, covering bar diameters of 8 mm, 16 mm, and 25 mm and inclination angles of 0 to 90° in 15° increments. The present work addresses the welding of segments within the SPI process, in which the thermal exposure and the weld bead arise from the same operation, and leaves the geometric process effects to the companion study.

To isolate the effect of thermal exposure on bond strength, a controlled laboratory study was conducted prior to the present work. Conventionally cast pull-out specimens with WAAM-fabricated reinforcing bars heated to 20 °C, 60 °C, 80 °C, and 200 °C by means of an internal cartridge heater were tested according to RILEM RC6 [26]. The results revealed a displacement-dependent thermal effect that runs counter to the expectation of a uniform bond reduction across all slip levels. At small slip values (0.001 mm and 0.01 mm), the 200 °C specimens exhibited higher bond stresses than those at 60 °C and 80 °C, attributed to heat-induced water evaporation at the steel–concrete interface, which locally reduces the water-to-cement ratio and increases matrix stiffness. The 200 °C specimens showed a reduction of 29% in maximum bond stress relative to the unheated reference at 20 °C (13.99 versus 19.67 MPa). Complementary microstructural analyses by X-ray diffraction (XRD), thermogravimetric analysis (TGA), and mercury intrusion porosimetry (MIP) confirmed partial dehydration of calcium silicate hydrate (C-S-H) phases, an increased content of unhydrated tricalcium silicate (C_3_S), which indicates a locally reduced degree of hydration due to premature water loss, and a shift of the pore size distribution towards larger radii at 200 °C [26].

These findings establish the thermal sensitivity of the bond interface under controlled conditions. Independent evidence from cyclic thermal loading tests on reinforced concrete beams (7, 14, 21, and 28 heating and cooling cycles with peak temperatures of 100 °C, 200 °C, and 300 °C) shows that lap-splice bond strength is close to or above that of the unheated control at 100 °C and 200 °C but is reduced by up to 44% at 300 °C, and that the effect of the thermal cycles generally increases with their number [27]. For oven-heated specimens, bond losses additionally increase with heating rate and target temperature [28], and depend non-monotonically on the concrete age at exposure for ages of 3 to 28 days [29]. The thermal exposure considered here, by contrast, acts within the first hours after intrusion, before the interface has fully formed. The controlled study used conventionally cast specimens with a cartridge heater to apply a defined, uniform temperature over the bond length. In the actual combined SPI-WAAM process, the thermal exposure differs in character: it arises from the layer-by-layer deposition of reinforcement directly in the particle bed, producing a transient, spatially non-uniform temperature field. The cement paste surrounding the reinforcement is not conventionally cast but formed by selective intrusion into a granular packing.

The preceding studies thus established the thermal limits of the SPI system and the sensitivity of the bond to sustained heating, but they used defined, uniform temperatures in conventionally cast specimens or did not include bond tests. None of them joined reinforcement by welding within the SPI process itself. The present study takes this step as a first, manual approximation of WAAM. During SPI printing, segments of 16 mm reinforcing bars embedded vertically in the particle bed were joined by manual gas tungsten arc welding, a stud-welding-like procedure that exposes the bond zone to repeated welding-induced heating and cooling cycles. The thermal exposure was recorded by thermography, and the bond was determined in push-through tests. As the reference without heat input, segments of the same bars were joined by an adhesive under the same printing conditions. Segmentation is required for both groups, since the bar can protrude above the particle bed only by a limited distance during printing without colliding with the print heads (Section 2.2). Based on the controlled study, the hypothesis was that the welding-induced thermal exposure reduces the bond capacity of the welded bars relative to the adhesively bonded reference, as observed after sustained heating to 200 °C [26]. Sixteen specimens were produced in two production series, eight welded and eight adhesively bonded.

## 2. Materials and Methods

### 2.1. Materials

The concrete was produced using ordinary Portland cement (CEM I 42.5 N, Heidelberg Materials, Heidelberg, Germany) and quartz sand (Quarzwerke, Frechen, Germany) with a particle size ranging from 1.0 to 2.2 mm and a median particle size $d_{50}$ of 1.6 mm. The water-to-cement ratio was 0.40. A polycarboxylate-ether-based superplasticiser (Master Builders Solutions, Trostberg, Germany) was added at 0.8% by mass of cement to adjust the flow properties of the cement paste, targeting a mini-slump flow of 400 to 410 mm, determined with a Haegermann cone following DIN EN 1015-3 [30] but without jolting. The cement paste fills the void volume of the particle bed, which corresponds to a volume fraction of 42% cement paste and 58% aggregate. Assuming complete intrusion and a cement density of 3100 kg m^−3^, this gives a cement content of approximately 580 kg m^−3^ and a water content of approximately 230 kg m^−3^. Ribbed reinforcing bars of grade B500B according to DIN 488-1 [31] with a nominal diameter of 16 mm were used. For this mixture, a mean compressive strength of 72.4 MPa at an age of 28 days was determined in a previous study on cast prisms (40 × 40 × 160 mm) according to DIN EN 196-1 [26,32].

### 2.2. Specimen Geometry and Reinforcement Configuration

**Figure 2:**
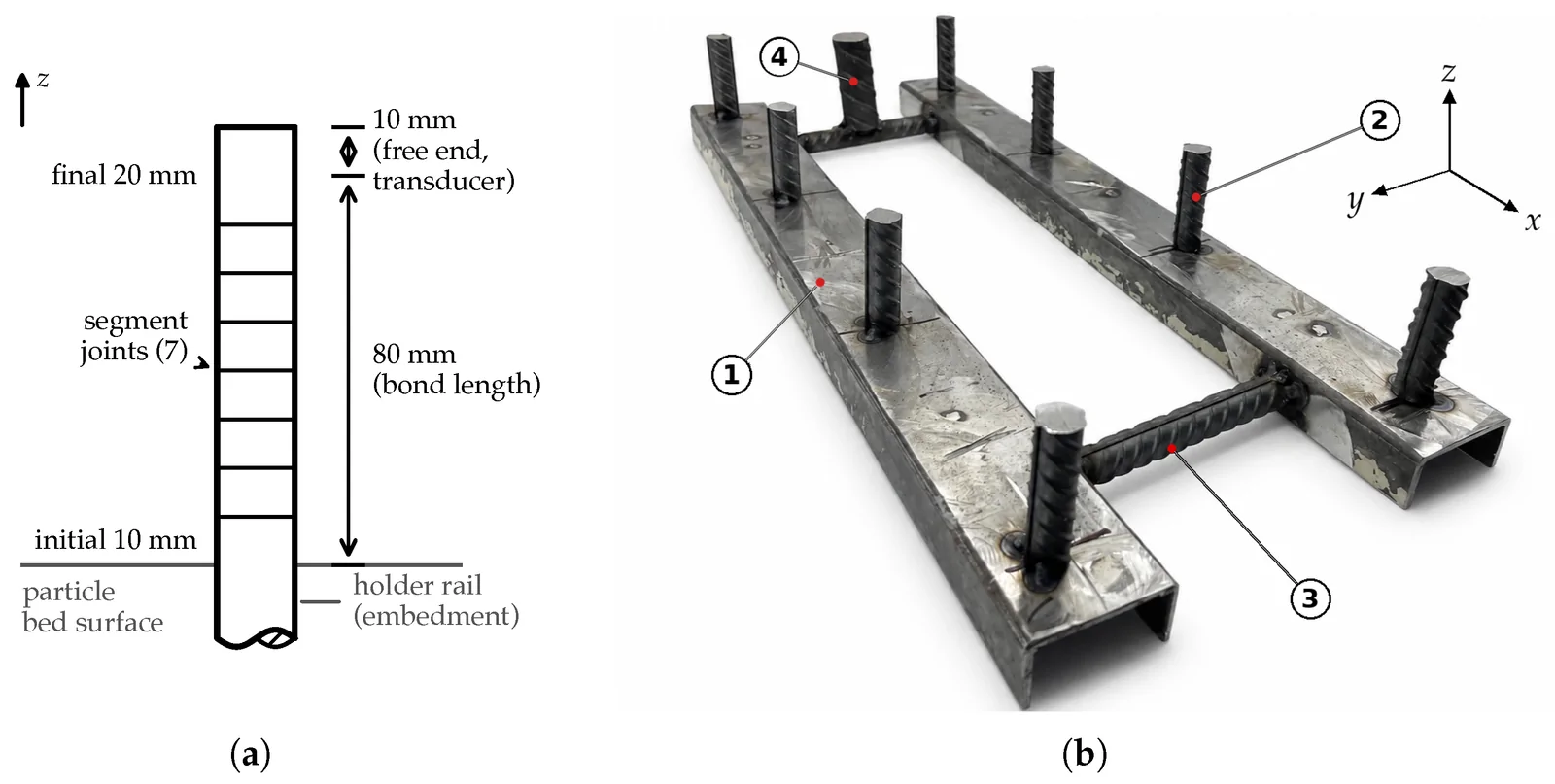
Specimen geometry and holder assembly. (**a**) Segment sequence and specimen geometry (to scale). (**b**) Holder assembly: (1) steel rails, (2) reinforcement segments, (3) transverse struts, (4) co-printed ground bar for the welding process.

The bond length was $5\;d_{s}=80$ mm. Each specimen started with an initial segment protruding 10 mm above the particle-bed surface. Below the bed surface, approximately 50 mm of bar extended down to the holder rail. After separation from the rail, this free length was the load-application end of the push-through test (see Section 2.4). Seven further segments were joined sequentially during printing, with the first six measuring 10 mm and the final segment measuring 20 mm, giving a total bar length of 90 mm above the initial bed surface (approximately 140 mm above the holder rail). Of this, 80 mm was embedded in the printed concrete (bond length), while the upper 10 mm of the final segment was left free above the specimen for mounting the displacement transducer. Seven segment joints fall within the bond length (Figure 2a). The reinforcing bars were cut into these segments prior to fabrication using a thin cutting disc, perpendicular to the bar axis. During fabrication, the segments were reassembled in the original cutting sequence and orientation using the respective joining method. Segmentation was required by the process. During printing, the bar grows with the particle bed and can protrude above the bed only within the clearance below the print heads, since a longer protrusion would collide with them. Print quality remains unimpaired only up to a nozzle distance of about 40 mm [3]. The bar of 90 mm above the initial bed surface therefore had to be built up from segments during printing, as in the companion study [25], and could not be placed in the particle bed as a continuous bar. The reference had to be segmented in the same way and had to remain free of heat input, so that the thermal effect of the welding could be assessed. Its segments were therefore joined by an adhesive at room temperature. Because the adhesive butt joints cannot transfer the tensile force of a pull-out test according to RILEM RC6 [33], both groups were tested in push-through, with the loading direction reversed and the same evaluation of bond stress versus slip (Section 2.4).

Two joining methods were compared. In the welded group, the segments were joined by gas tungsten arc welding (GTAW) using a power source (REHM Invertig.Pro digital 240DC, Uhingen, Germany) with an air-cooled torch (R-Tig 200/35) and a steel filler rod (W3Si1 according to DIN EN ISO 636 [34]) under a pure argon (I1 according to DIN EN ISO 14175 [35]) shielding gas atmosphere. The welding was performed in direct current electrode negative (DCEN) polarity at a machine-set welding current of 120 A with a filler rod diameter of 1.0 mm. Torch and filler rod were guided manually, as is characteristic of manual GTAW, so that arc duration and filler addition varied from joint to joint. In each joining cycle, one segment was welded onto each bar of the welded rail in the order P01 to P04 and one onto the ground bar. Consecutive bars were welded about 1.1 min apart, measured between the Δ*T* peaks of P02 to P04 (Section 2.5). The mean interval between the joining cycles was 17.6 and 15.4 min in series 1 and 2. All welds were made by the same operator. Prior to each welding operation, the exposed bar end and the joint faces were cleaned to remove adhering aggregate particles, dust, and cement paste from the particle-bed environment. In the adhesively bonded group, the segments were butt-joined using a low-viscosity ethyl cyanoacrylate adhesive (Loxeal Instant Adhesive 32, Cesano Maderno, Italy). The joint faces of this group were cleaned prior to joining in the same manner as those of the welded group.

The reinforcing bars were fixed in the particle bed by welding them onto a holder consisting of two steel rails. Each rail carried four bars at a spacing of approximately 180 mm along the rail, and the two rails were mounted approximately 180 mm apart. The rails were connected by transverse steel struts for stability. A vertical ribbed bar (diameter 28 mm) was the ground connection for the welding process. This bar was co-printed with the specimens and segmented in the same manner as the reinforcing bars, so that a fresh segment was always available above the bed surface for attaching the welding clamp. Figure 2 shows the specimen geometry and the holder assembly.

### 2.3. Specimen Fabrication

The specimens were fabricated on a laboratory SPI printer with a build volume of 600 × 350 × 350 mm. The aggregate was spread in layers of 3 mm thickness using the spreading roller of the printer, followed by a doctor blade. After each aggregate layer, cement paste was selectively applied by two print heads with eight nozzles each (nozzle diameter 1 mm) at a print speed of 300 mm s^−1^ and a nozzle distance of 20 mm above the bed surface. Assuming that the paste fills the void volume of the layer, the paste quantity was approximately 1.3 L m^−2^ of printed area per layer. Without segment joining, one layer took about 55 s. The printing took place at (20 ± 1) °C and a relative humidity of 44 to 50%. For printing, the holder was placed on the printer platform and both rails were overprinted simultaneously in the same printing sequence, so that the welded and adhesively bonded groups received identical cement paste and aggregate conditions.

The printing started with the first 10 mm segment protruding above the particle bed. With each successive 3 mm layer of aggregate and cement paste, the protrusion decreased until the upper end of the segment approached the level of the bed surface. The printer was then paused, and a new segment was joined to the existing bar using the respective joining method (see Section 2.2), restoring the protrusion. This cycle of printing and segment joining was repeated until the full bond length of 80 mm was reached.

**Figure 3:**
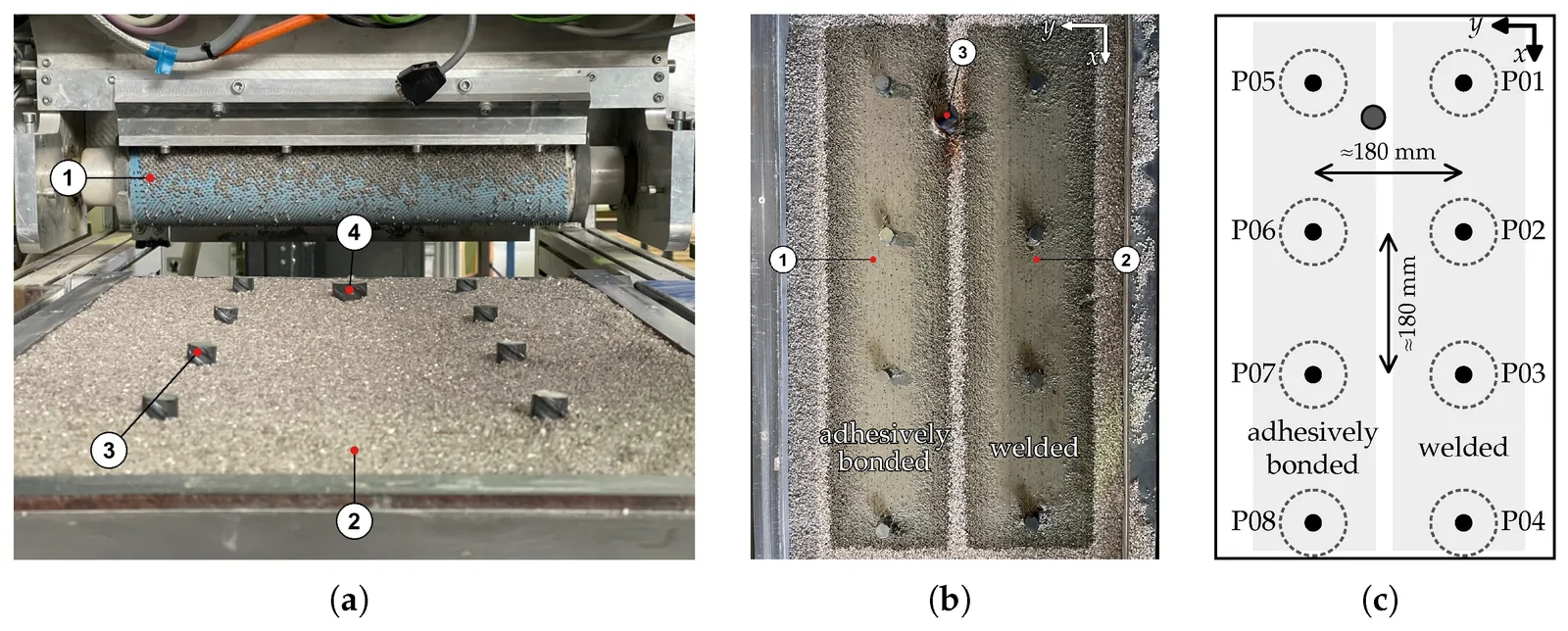
Specimen fabrication. (**a**) Holder assembly in the particle bed before printing: (1) spreading roller, (2) particle bed, (3) initial segments protruding 10 mm above the bed surface, (4) ground bar. (**b**) Top view after printing: (1) printed block along the adhesively bonded rail, (2) printed block along the welded rail, (3) ground bar. (**c**) Bar positions P01 to P04 (welded) and P05 to P08 (adhesively bonded) in the particle bed shown in (**b**), at the same scale, drill cores dashed, ground bar grey.

The setup produced a continuous block of printed concrete along each rail, embedding all four specimens per rail in a single monolithic body. This fabrication was performed in two production series of identical configuration, so that eight specimens per group were obtained. In both series, the adhesively bonded segments were mounted on the left and the welded segments on the right rail (Figure 3b). The bar positions are designated P01 to P04 on the welded rail and P05 to P08 on the adhesively bonded rail (Figure 3c), and the series is added as a suffix, for example P03.2 for position P03 in series 2. Both series used the same materials, mix design, and printing parameters.

After 24 h of curing in the particle bed, the blocks together with their holders were excavated. Since the embedded bars still connected the blocks to the holder rails, the reinforcement stubs were separated directly upon excavation to release the blocks for storage. The stubs were cut with a rotary tool (Dremel, Mount Prospect, IL, USA) and a thin cutting disc to limit vibration-induced loading of the early-age bond at the steel–concrete interface. The blocks were then stored at 20 °C and 65% relative humidity.

Since the SPI process produces a monolithic block rather than individual cylindrical specimens, each specimen had to be isolated by core drilling. At an age of 21 days, four cylindrical specimens with a diameter of 80 mm were extracted from each block by wet core drilling, with the reinforcing bar centred within each core. The concrete cover of 32 mm (2 *d*_s_) remaining after core drilling is less than half of the cover of the RILEM RC6 specimen, whose cube edge length of 10 *d*_s_ gives a cover of $4.5\;d_{s}$ [33]. Without additional confinement, splitting failure therefore had to be expected. To provide the confinement required for a push-through test governed by bond failure rather than splitting, the specimens were bonded into steel confinement rings (inner diameter 82 mm, inner surface sandblasted to improve adhesive bond) using epoxy adhesive (Hilti HIT-RE 500, Schaan, Liechtenstein). Excess concrete above and below the ring was removed with a fine saw, producing specimens with a height equal to the bond length of 80 mm.

All specimens were stored at 20 °C and 65% relative humidity until testing at an age of 28 days in both production series. Figure 3 shows the key stages of the fabrication process.

### 2.4. Mechanical Testing

**Figure 4:**
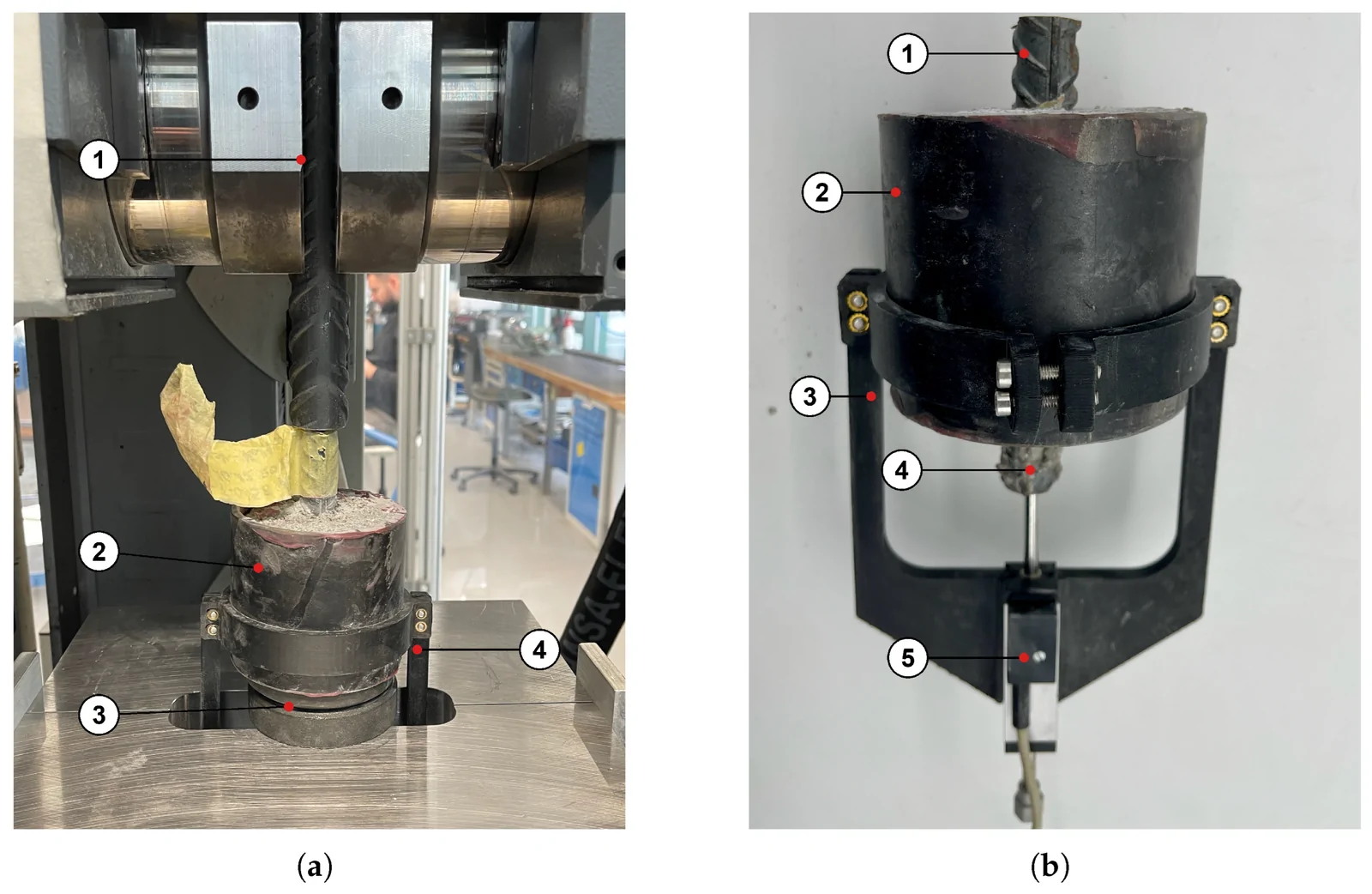
Push-through test configuration, as in the companion study [25]. (**a**) Specimen in the testing machine: (1) loading bar clamped in the grips, pressing on the protruding 16 mm bar, (2) specimen bonded into the steel confinement ring, (3) spherical seat, (4) transducer holder. (**b**) Instrumented specimen: (1) loaded bar end, (2) steel confinement ring, (3) polymer holder clamped to the ring, (4) free bar end, (5) linear displacement transducer.

Push-through tests were conducted following a procedure adapted from RILEM RC6 [33], as in the companion study [25] (Figure 4). The specimens were placed on a spherical seat to compensate for angular misalignment between bar axis and load axis and to ensure axial load application. The reinforcing bars protruded vertically upwards, and the specimens were tested inverted relative to the printing orientation: the former rail-side bar end pointed upwards for load application, while the displacement transducer engaged the opposite bar end (the final segment) below the specimen. Force-controlled loading was applied at the loading rate of RILEM RC6 [33], which was adopted for comparability. It was computed as $\dot{F}=0.5\;N\;s^{-1}\;{\mathrm{mm}}^{-2}\;\cdot \;d_{s}^{2}=128\;N\;s^{-1}$ for $d_{s}=16\;\mathrm{mm}$. Load was applied to the upper bar end on a universal testing machine (Zwick Z600, Ulm, Germany). Force was recorded using the internal load cell of the testing machine.

Displacement was recorded at the free (unloaded) bar end using a potentiometric linear displacement transducer (Ahlborn FWA025TR, Holzkirchen, Germany, measuring range 25 mm, resolution 0.001 mm as specified by the manufacturer) mounted in a polymer holder clamped to the steel confinement ring. Because the displacement is measured at the free end, it does not contain the elastic shortening of the bar or the deformation of the segment joints between the loaded and the free end, in the same way as the free-end slip of a pull-out test according to RILEM RC6 [33] does not contain the elastic elongation of the bar. The bond stress τ was calculated from the applied force *F* as

(1) $$\tau =\frac{F}{\pi \;\cdot \;d_{s}\;\cdot \;l_{b}}$$

with $d_{s}=16$ mm and $l_{b}=80$ mm. Equation (1) corresponds to the mean bond stress over the bond length as defined in RILEM RC6 [33], applied here without the normalisation to a reference concrete strength used there. The bond response was evaluated at four characteristic slip levels: τ(0.001), τ(0.01), τ(0.1), and $\tau_{\max}$ (maximum bond stress, referred to as bond capacity in the following), representing the adhesive stage, the early interlock stage, the developed interlock stage, and the bond capacity, respectively. This evaluation follows the practice of the preceding studies [7,26] and reflects the stages of bond action described in fib Bulletin 10 [13].

The configuration deviates from RILEM RC6 [33] in several respects. Loading is applied in compression rather than tension (Section 2.2), so that lateral expansion of the bar due to the Poisson effect increases the radial pressure at the interface instead of reducing it. Over the full specimen height, the bar is bonded, without the bond-free pre-length of the RC6 specimen. The concrete cover of the RC6 cube is replaced by a steel confinement ring, whose radial stiffness exceeds that of a typical concrete cover. Finally, the concrete is built up layer-wise along the bar axis rather than cast transverse to it. The absolute bond stresses reported here therefore characterise this confined push-through configuration, and the two groups are compared within it. Both groups were fabricated and tested in the identical configuration, but the configuration can interact with the joint type, since the entire load is transferred through the segment joints in compression (Section 3.4). The RC6-derived bond length, loading rate, and slip-level evaluation were retained for comparability with the preceding controlled study [26] and with the pull-out characterisation of WAAM reinforcement [7].

### 2.5. Thermographic Imaging

Infrared video was recorded during each production series using a thermal imaging camera (VarioCAM Research 976S, InfraTec, Dresden, Germany, long-wave infrared 8 to 14 μm) with a 30 mm objective (Jenoptik, Jena, Germany). From a distance of approximately 1.1 m, the camera looked vertically down onto the particle bed. The emissivity was set to ε=1.00, the reflected temperature to 20 °C, and the calibration range was −40 to 120 °C. The video was exported at 1024×768 pixels and 15 frames per second. Derived from the bar spacing of about 180 mm on the rail, the image scale was approximately 0.6 mm per pixel. It changed by up to 7% during a recording, because the bed surface rose towards the fixed camera. The emissivity setting approximates the emissivity of the coarse quartz sand surface, which is about 0.9 in the long-wave infrared [36,37,38], and was chosen because the primary objective was to monitor the temperature distribution in the particle bed. For the steel surface, the displayed temperatures are too low, because freshly cut steel emits far less radiation ($\varepsilon_{\mathrm{steel}}\approx$ 0.20 to 0.30). An approximate steel-surface temperature was therefore estimated by grey-body inversion over the camera band of 8 to 14 μm with a reflected temperature of 20 °C. For ε= 0.20 to 0.30, a displayed 100 °C corresponds to approximately 214 to 277 °C and a displayed 60 °C to approximately 125 to 162 °C. Because the steel surface oxidises over successive welding cycles and its emissivity increases, the actual temperatures may be lower. Relative to the estimate for ε=0.30, an emissivity of 0.4 lowers the estimated temperature rise above 20 °C by about 19%, one of 0.6 by about 40%, and one of 0.8 by about 52%. A reflected temperature of 15 °C instead of 20 °C raises the estimates by up to 8 K, and one of 30 °C lowers them by up to 19 K. The temperatures were reconstructed from the colour-coded video frames by assigning each pixel the temperature of the nearest colour of the displayed temperature scale. Changes of the displayed scale range during a recording were bridged by cross-calibration on frames extracted at 5 s intervals. For the quantitative evaluation, frames were extracted at 30 s intervals.

**Figure 5:**
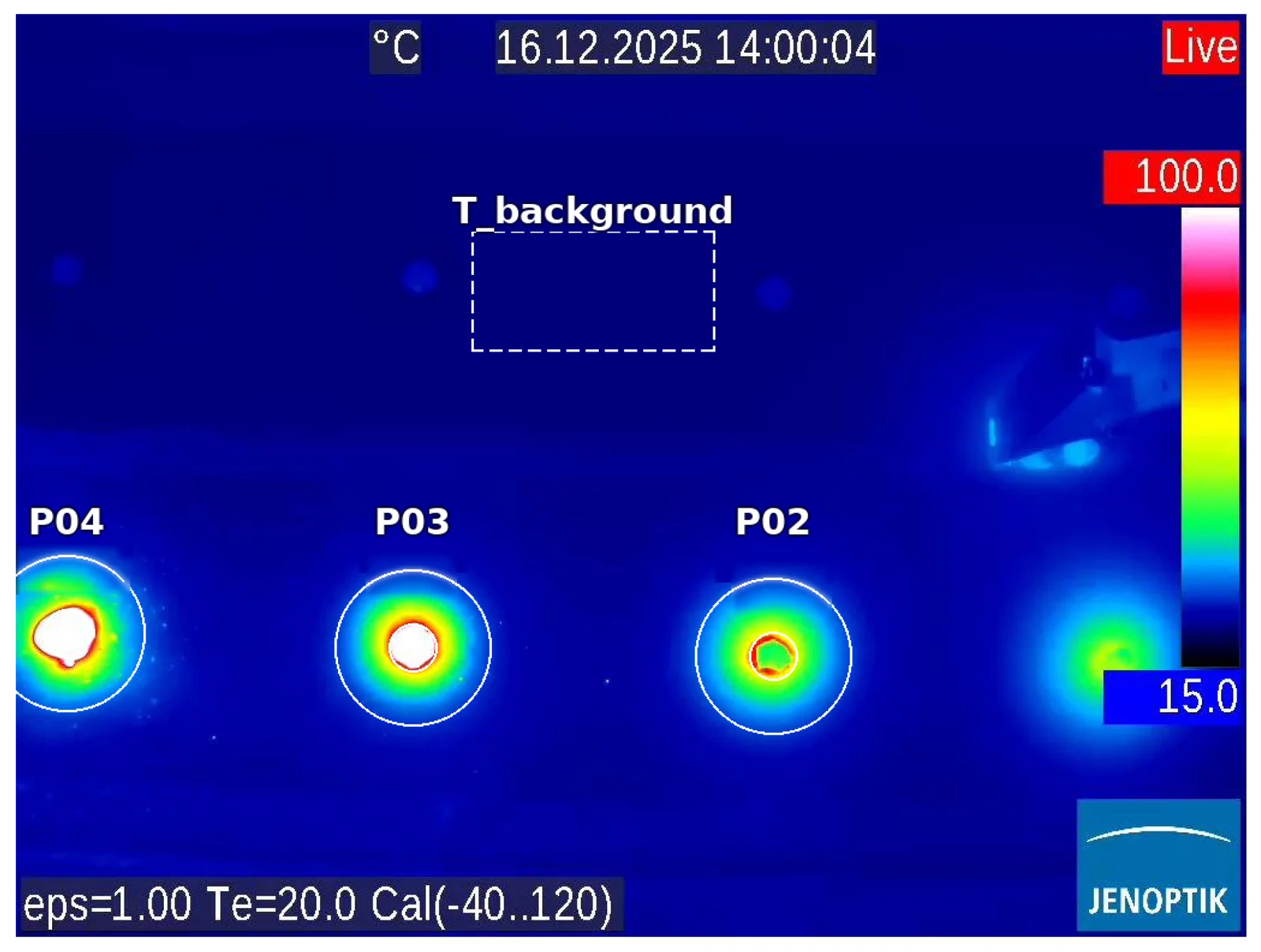
Exemplary infrared frame with the evaluation regions overlaid. The annular ROI (white rings) surrounding each of the three evaluated bars (P02, P03, P04) defines $T_{\mathrm{halo}}$. The rectangular reference area (dashed outline) in a bar-free region of the same frame defines $T_{\mathrm{background}}$.

From each frame, the excess temperature $\Delta T=T_{\mathrm{halo}}-T_{\mathrm{background}}$ was computed. $T_{\mathrm{halo}}$ is the mean temperature in an annular region of interest (ROI) around each bar with an inner radius of 20 pixels (approximately 12 mm), which excludes the bar cross-section, and an outer radius of 65 pixels (approximately 39 mm), giving about 12,000 pixels per ROI. The ROI geometry was identical for all bars and recordings. The bar positions were tracked from frame to frame by centroid detection. They drifted by up to about 25 pixels over a recording, and an evaluation with fixed positions changed the integrals by less than 2%. $T_{\mathrm{background}}$ is the mean temperature in a rectangular bar-free reference area of the same frame. This frame-by-frame background correction eliminates the baseline drift caused by periodic non-uniformity corrections (NUC) of the camera sensor and the uncertainty of the absolute temperature reconstruction of about 5 K. Because both quantities are extracted from the same frame with the same emissivity setting, the ΔT values are largely independent of the absolute emissivity calibration. Three bars per recording (P02, P03, P04) were evaluated. P01 was excluded because the overlay of the temperature scale prevented a complete annular ROI. Figure 5 shows an exemplary frame with the evaluation regions overlaid. The cumulative thermal exposure was quantified as the time integral ∫max(ΔT,0)dt, evaluated by the trapezoidal rule on the 30 s series from the start of the recording until 5 min after the last welding peak, for each bar and for the mean curve of the three bars of a recording.

### 2.6. Data Evaluation

All results are reported as group means with standard deviations (SD) and coefficients of variation (CV), and the individual values are listed in Appendix A (Table A1). The joining method was assigned to a complete holder rail. The four specimens of a rail therefore share the printed block, the curing, and, in the welded group, the welding sequence of the series, so that they are not fully independent of each other. To quantify the uncertainty of the comparison, the difference between the group means (welded minus adhesively bonded) is given with its 95% confidence interval, calculated with Welch’s *t*-test for unequal variances. The interval contains the differences that are compatible with the measured scatter. If it includes zero, no difference is detected, and its lower bound indicates the largest reduction that the data cannot exclude. Because the specimens of a rail share the production, the difference between the groups is also reported separately for each series.

## 3. Results and Discussion

### 3.1. Bond Behaviour

**Figure 6:**
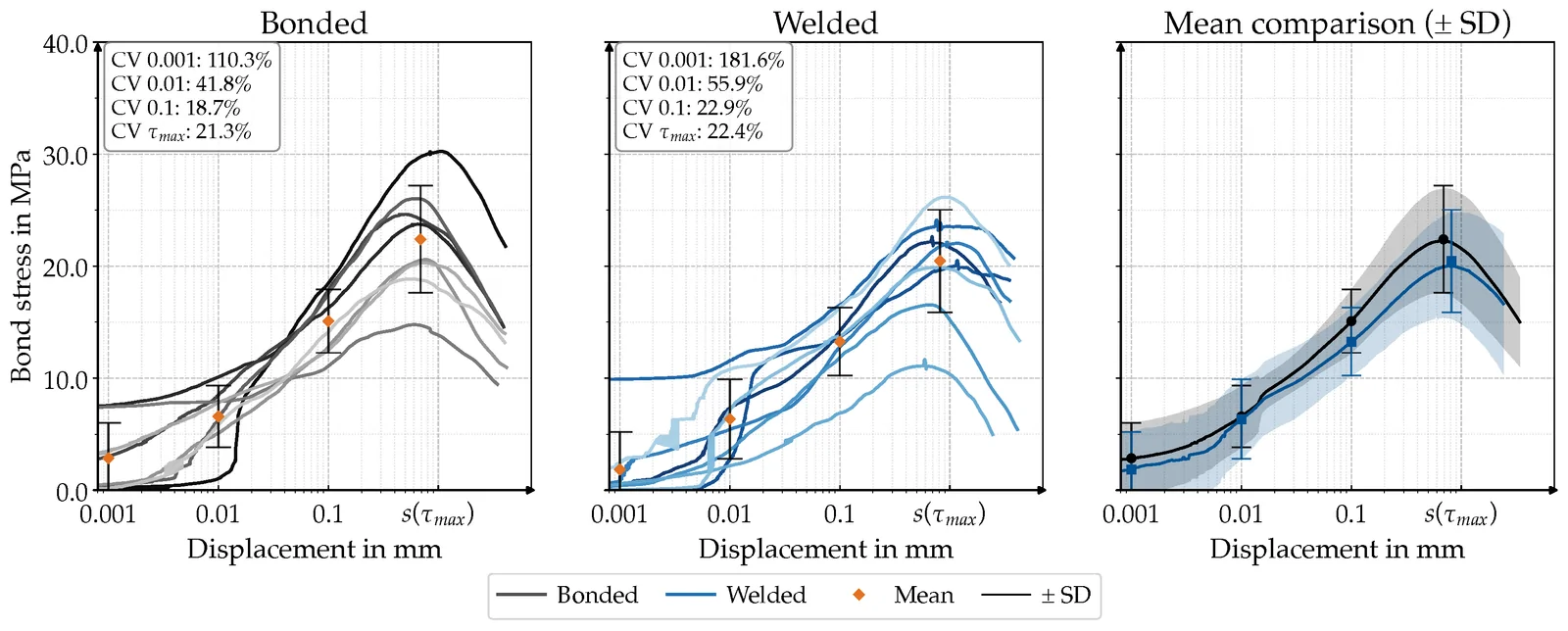
Individual bond-stress–displacement curves (continuously measured) for the adhesively bonded (**left**, labelled “Bonded”) and welded (**centre**) specimens, with mean comparison (**right**). The fourth axis tick, labelled $s(\tau_{\max})$, marks the maximum-bond-stress level. Mean displacements at maximum bond stress are 0.69 mm (adhesively bonded) and 0.81mm (welded). Each mean curve is truncated at the largest displacement reached by all specimens.

**Figure 7:**
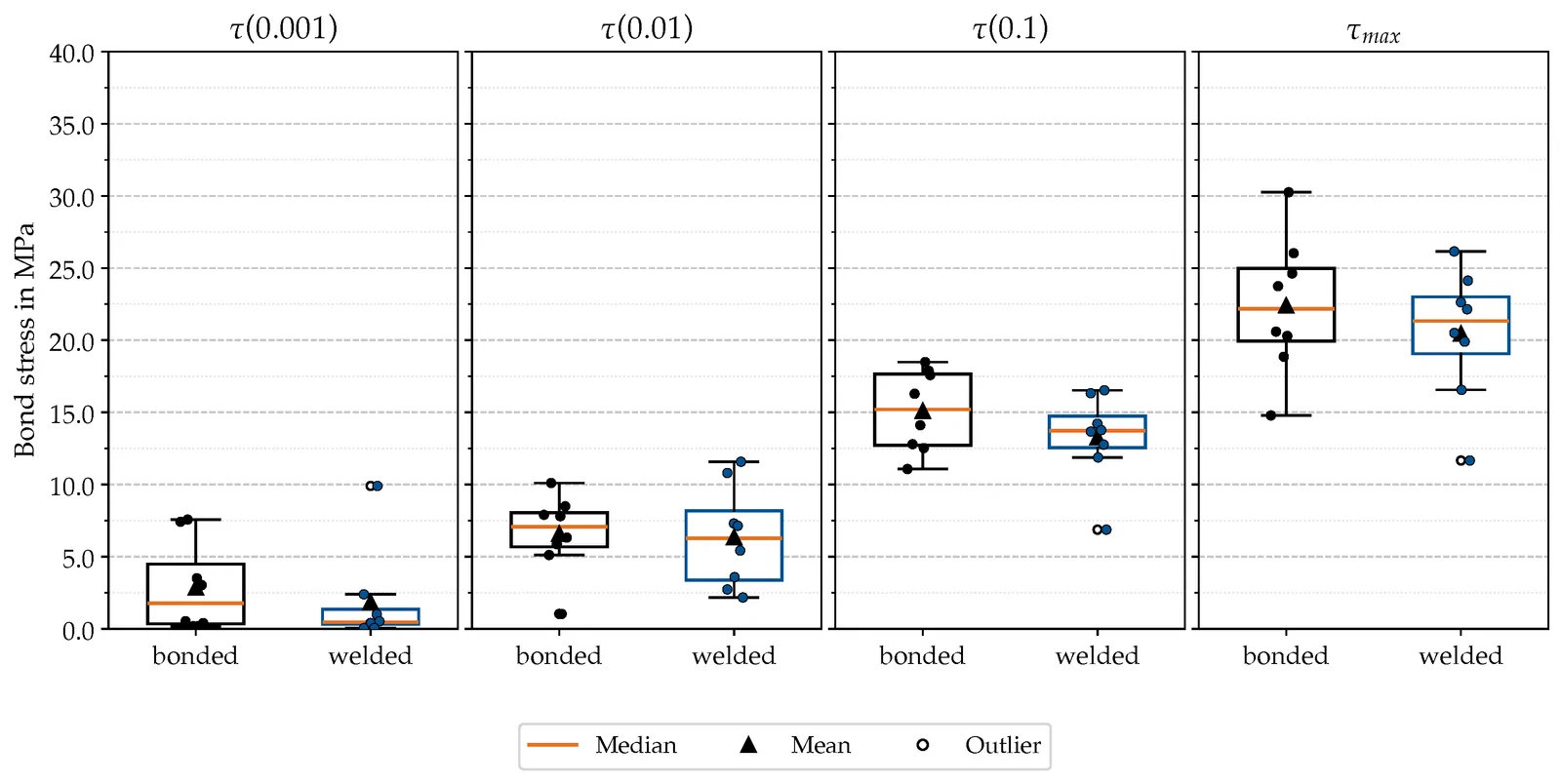
Box plots of the discrete bond stress values at s=0.001 mm, s=0.01 mm, s=0.1 mm, and at $\tau_{\max}$ (n=8 per group), with “bonded” denoting the adhesively bonded group. Open circles mark outliers beyond 1.5 times the interquartile range.

Figure 6 shows the individual bond-stress–displacement curves for the welded and adhesively bonded specimens, together with the mean comparison. Figure 7 compares the discrete bond stress values of the two groups at s=0.001 mm, s=0.01 mm, s=0.1 mm, and at $\tau_{\max}$. All 16 specimens (n=8 per group) are included in the analysis. Table 1 summarises the bond stresses at the four characteristic slip levels, and Table A1 in Appendix A lists the individual values.

All curves follow the characteristic sequence of adhesion-controlled initial response, progressive activation of mechanical interlock at the ribs, attainment of a peak bond stress, and subsequent frictional decay, consistent with the classical bond behaviour reported for ribbed bars [12,13,14]. No specimen failed by splitting. All failures occurred by bar push-through. The measured response is thus governed by bond failure.

At small displacements (s=0.001 mm and s=0.01 mm), the two groups show no consistent difference. The mean values are 1.8 MPa (welded) and 2.9MPa (adhesively bonded) at s=0.001 mm, and 6.3 MPa and 6.6 MPa at s=0.01 mm. The scatter at these displacement levels is high in both groups (CV at τ(0.001): 182% welded, 110% adhesively bonded), since displacements this small approach the resolution limit of the displacement measurement and are sensitive to local packing and intrusion conditions. These levels are therefore reported for completeness.

The curves diverge from approximately s=0.05 mm onwards. At s=0.1 mm, the adhesively bonded group reaches a mean bond stress of 15.1 MPa, compared with 13.3 MPa for the welded group.

At $\tau_{\max}$, the welded specimens achieve a mean of 20.5 MPa (SD = 4.6 MPa) and the adhesively bonded specimens 22.4 MPa (SD = 4.8 MPa). The difference of 1.9 MPa, corresponding to 9% of the adhesively bonded mean, is smaller than the standard deviation of either group, and the coefficients of variation are 22% (welded) and 21% (adhesively bonded). Within the series, the welded specimens reach 3.8 MPa (series 1) and 0.1 MPa (series 2) less than the adhesively bonded specimens (Table A1). No discernible differences were therefore detected in this sample and under this experimental condition. The 95% confidence interval of the difference ranges from −7.0 to 3.1 MPa. A reduction of up to 7 MPa, or 31% of the adhesively bonded mean, therefore cannot be excluded with eight specimens per group from two production series.

The slip at $\tau_{\max}$ is 0.81 mm (welded) and 0.69 mm (adhesively bonded), a difference of 0.12 mm with a 95% confidence interval of −0.07 to 0.32 mm.

The scatter of both groups (CV 22% welded, 21% adhesively bonded) exceeds the range reported for pull-out testing of ribbed bars. Compilations of nominally identical pull-out specimens report coefficients of variation of 1 to 18% [39], individual 16 mm pull-out series reach 10.9% [40], and a standard deviation of about 1.3 MPa is reported for pull-out tests under ideal conditions [12]. The present specimens were manually segmented, reassembled, and joined, and they were extracted from printed blocks by core drilling rather than cast in standardised moulds.

The scatter decreases from τ(0.001) (CV 182% welded, 110% adhesively bonded) to $\tau_{\max}$ (22% and 21%). The standard deviations change little between the slip levels (2.7 to 4.8 MPa), so that the decrease of the coefficient of variation follows from the increase of the mean bond stress, as reported for pull-out tests [12].

The mean bond capacities correspond to 28% (welded) and 31% (adhesively bonded) of the mean compressive strength of 72.4 MPa determined on cast prisms of the same mixture (Section 2.1). For SPI with an aggregate of the same particle size and a cement paste with a water-to-cement ratio of 0.30, printed specimens reached compressive strengths at 7 days equal to or above those of cast specimens of the same density at 28 days, and showed no dependence on the loading direction relative to the layers [2]. The prism strength can therefore be regarded as a conservative estimate for the printed matrix.

### 3.2. Thermal Exposure

**Figure 8:**
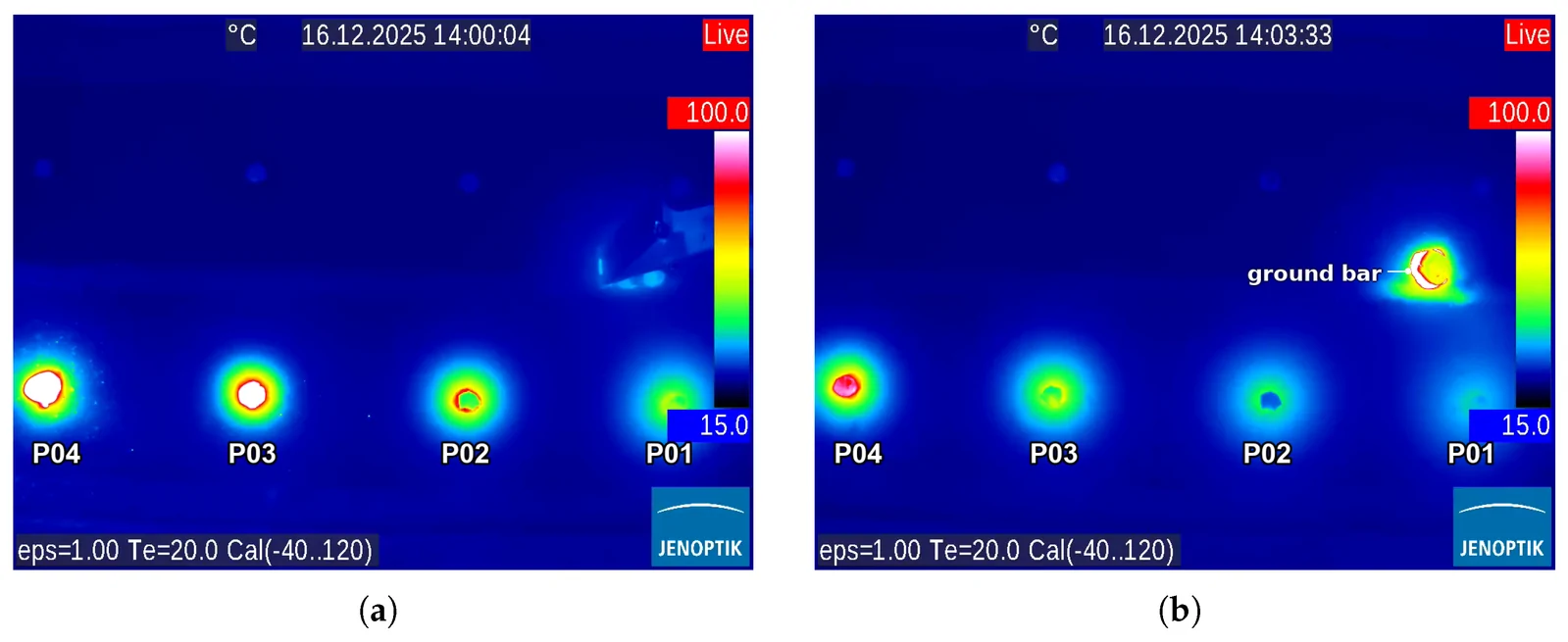
Representative infrared images of a segment-joining cycle, with displayed temperatures at ε=1.00 (Section 2.5). (**a**) Directly after welding of P04, with thermal halos of the preceding welds at P01 to P03. (**b**) About 3.5 min later, with the co-printed ground bar marked. The adhesively bonded rail (upper part of the image) shows no thermal signature.

In the infrared images (Figure 8), the region around the welding point exceeds a displayed temperature of 100 °C. Displayed temperatures are the camera readings at ε=1.00. For the particle bed they are close to the actual temperature, for the steel surface they are too low (Section 2.5). For the bar surface, a displayed 100 °C corresponds to an estimated 214 to 277 °C for ε= 0.20 to 0.30, and to lower values if the surface has oxidised (Section 2.5). These estimates are of the same order as the bar temperatures of 164 to 206 °C measured by thermocouples at 40 mm from the process zone in a preceding study [24]. These values refer to the bar surface in the particle bed near the joint, not to the peak temperatures in the weld pool and the heat-affected zone, which are higher. The bars cool from >100 °C (displayed) to approximately 60 °C (displayed, estimated 125 to 162 °C) within 2 to 3 min. Over the course of the fabrication, the thermal halos of adjacent bars on the welded rail become visible on the particle-bed surface, which shows that heat is transferred into the surrounding particle bed. As the particle bed is progressively covered by fresh aggregate and cement paste during printing, the surface measurement captures the thermal conditions at the time of deposition rather than a continuous record of the temperature at the steel–concrete interface. The adhesively bonded rail shows no thermal signature at the bed surface at any point during fabrication.

At the welding peaks, the excess temperature at the bed surface decreased with the distance from the bar centre to about 3 to 6 K at 40 mm, the radius of the drill cores, and fell below 2 K at about 50 mm (evaluated at four welding peaks with the image scale of Section 2.5). This estimate is based on the successively covered bed surface, i.e., a two-dimensional projection of the heat propagation.

**Figure 9:**
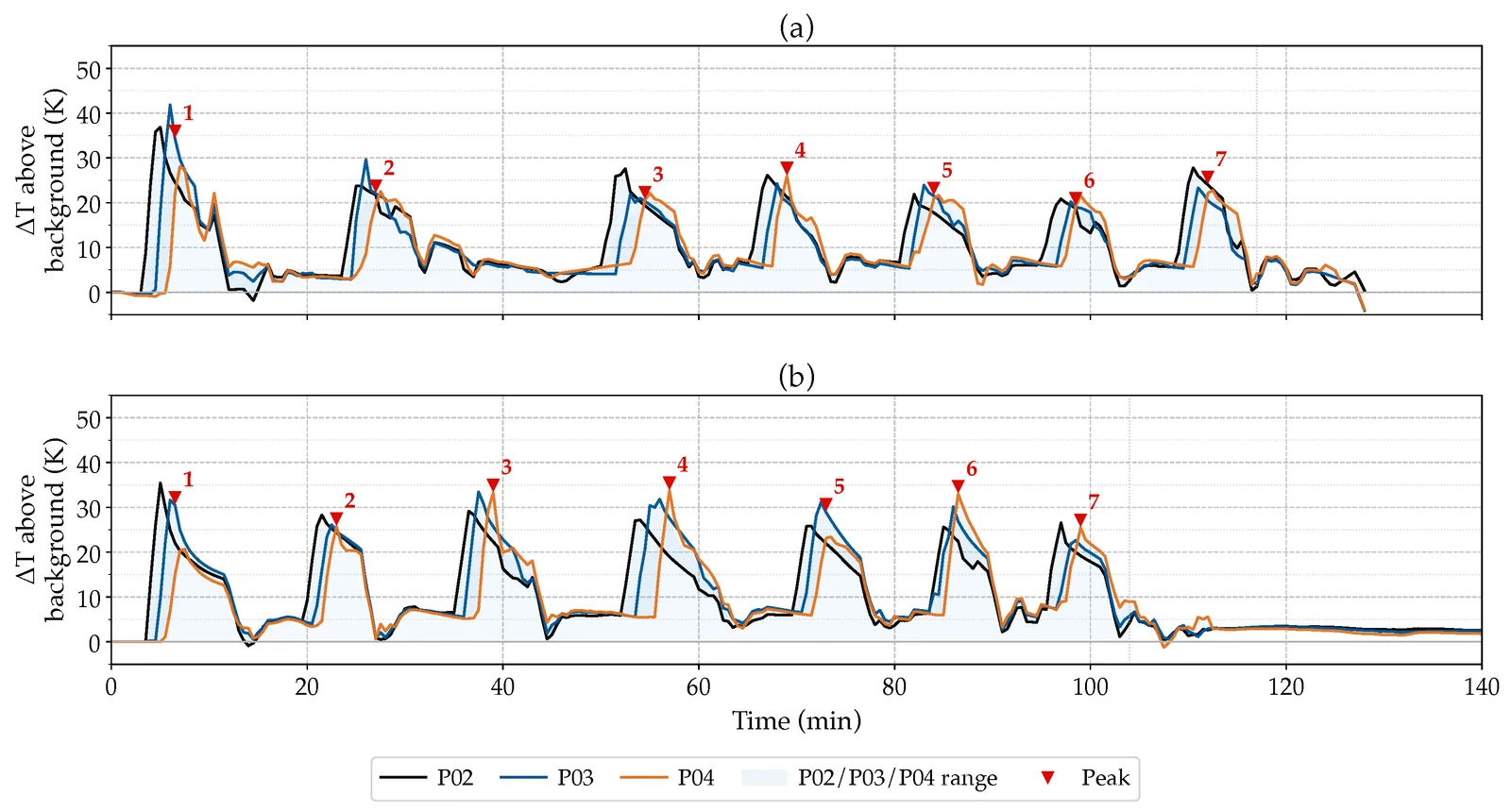
Excess temperature ΔT of the particle-bed surface around the welded bars P02, P03, and P04 in series 1 (**a**) and series 2 (**b**), with the range between the bars shaded and the welding peaks of the mean curve marked by triangles above it and numbered 1 to 7 in the order of the joining cycles. ΔT is evaluated on the particle bed, for which the camera setting ε=1.00 is close to the actual emissivity. The negative values at the end of series 1 are a background-correction artefact outside the evaluation window.

The quantitative ΔT evaluation (see Section 2.5) shows seven distinct peaks per recording, each corresponding to one segment-joining cycle, during which one segment was welded onto each of the four bars and the ground bar in direct succession (Figure 9). Each cycle follows a characteristic sequence: a steep temperature rise during the arc phase, a gradual decay as heat disperses into the surrounding particle bed, a drop when the next aggregate and cement paste layers are spread over the heated zone, followed by a slight temperature recovery as stored heat from the bed re-enters the freshly deposited material. This recovery indicates that the particle bed stores heat transiently and extends the effective duration of the thermal exposure beyond the welding event itself.

The mean peak values are $\Delta T_{\mathrm{peak}}=21.7$ K in series 1 and 25.1 K in series 2. Between the welding cycles, the mean excess temperature fell to 0.4 to 4.0 K, so that the visible particle-bed surface cooled close to ambient. The mean intervals between the joining cycles were 17.6 and 15.4 min, because the joining was performed manually.

The cumulative thermal exposure at the bed surface, quantified as the time-integrated excess temperature ∫max(ΔT,0)dt of the mean curve, amounts to 1165 K min in series 1 and 1212 K min in series 2. For the individual bars P02, P03, and P04, the integrals are 1260, 1145, and 1093 K min in series 1 and 1250, 1277, and 1109 K min in series 2. Within each series, they span 167 and 168 K min, which is more than three times the difference between the two series (47 K min, about 4%).

The thermography records the visible particle-bed surface, which is progressively covered by aggregate and paste. Its emissivity changes as the surface changes from dry quartz sand to intruded paste, and the bars are embedded below this surface. Because the heat flows from the bar into the bed, the steel–concrete interface reaches higher temperatures than the evaluated annulus at 12 to 39 mm from the bar centre. The integral of ΔT therefore characterises the thermal exposure of the deposition zone, not the cumulative thermal dose at the interface. The process-integrated exposure also differs from the controlled study [26], in which 12 mm WAAM bars were held at 200 °C for 18 min by an internal cartridge heater and showed a reduction of 29% in maximum bond stress. Here, high bar-surface temperatures act only for a few minutes per welding cycle, and the bed surface cools close to ambient between the cycles. Tests on hardened concrete at sustained temperatures of 150 to 350 °C show a bond degradation that depends mainly on the temperature level and increases with the heating duration [41]. The smaller mean difference in the present configuration is consistent with the lower temperature level and the shorter duration of the exposure.

### 3.3. Weld Geometry, Heat-Affected Zone, and Interface Microstructure

**Figure 10:**
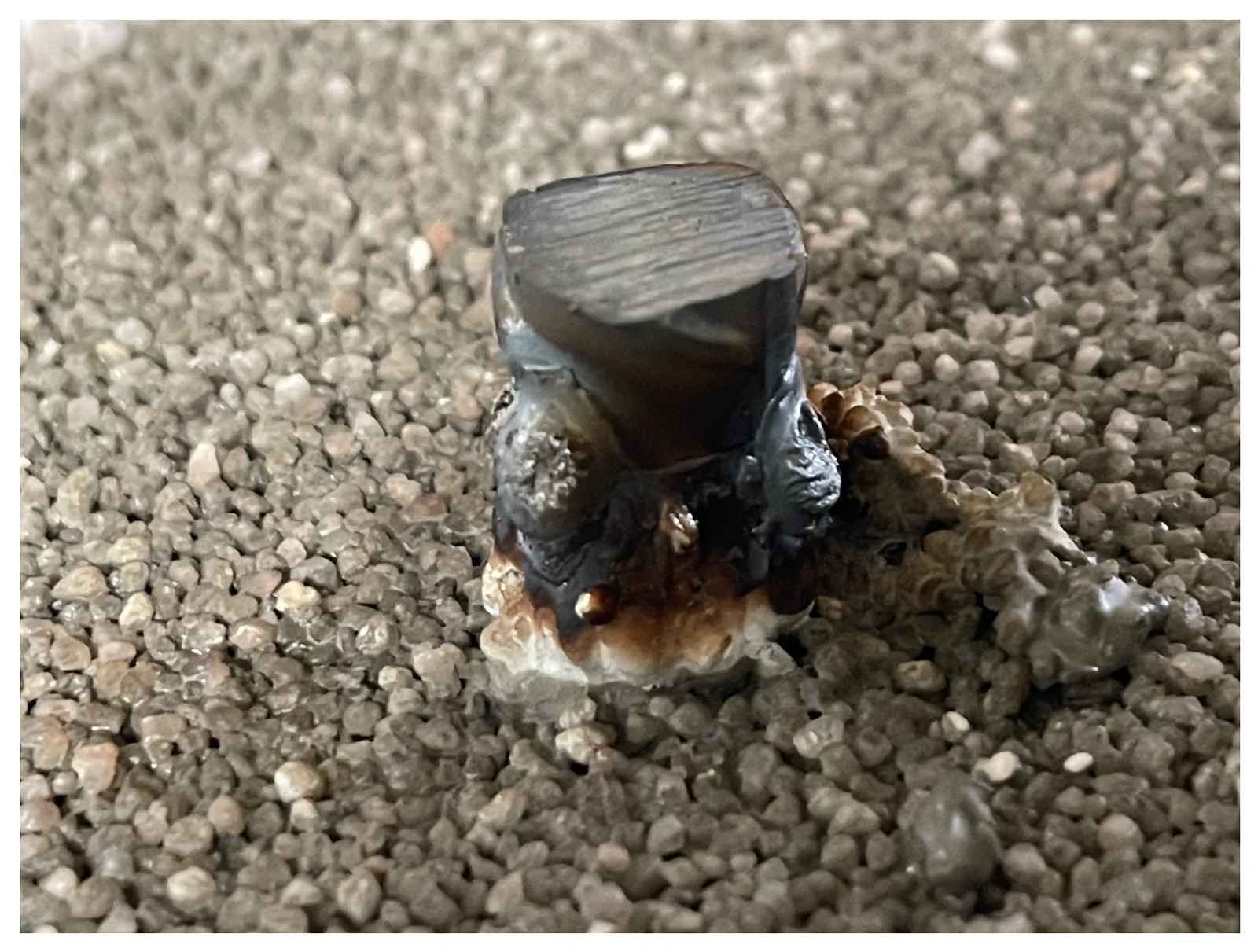
Close-up of a segment joint during fabrication, taken directly after a welding cycle. The weld bead protrudes from the bar surface, forming a localised geometric discontinuity.

The adhesively bonded segments are the reference without heat input that can be produced under the same SPI conditions, since segmentation is required by the process (Section 2.2). However, they are not an ideal unheated counterpart of the welded bar. Each welded joint carries a weld bead that protrudes from the bar surface (Figure 10) and may act as an additional bearing element, whereas the adhesively bonded butt joints are flush with the bar surface and contain a thin adhesive layer. The joints also differ in axial stiffness and continuity. A thermally induced bond reduction could therefore be partly offset by mechanical interlock at the weld beads, and the comparison reflects the welding procedure as a whole, in which the thermal exposure cannot be fully separated from the joint geometry. For the joining of reinforcement segments by welding during printing, this combined effect is the one that acts, since heat input and weld bead arise from the same operation.

Three joint-related mechanisms may influence the bond of the welded bars. First, the weld bead changes the bar surface at each joint and may act as a bearing element in addition to the ribs. Second, the heat-affected zone may have changed the steel properties and the rib geometry close to each joint. Because the joints were loaded in compression in the push-through test, such changes are more relevant for the tensile and fatigue performance of the joints than for the present bond results (Section 3.4). Third, the matrix at the interface close to the joints was exposed to the highest temperatures. After sustained heating to 200 °C, the controlled study found the changes in hydration and pore structure summarised in Section 1 [26]. In the present process, the short, repeated exposure together with the weld beads did not lead to a discernible bond reduction (Section 3.1).

WAAM-fabricated reinforcement, whose entire surface consists of successive weld beads, achieves bond performance comparable to conventionally ribbed B500B bars [7]. Bond degradation after elevated-temperature exposure of hardening or hardened concrete has been reported in multiple independent studies [27,28,29,42,43,44], and the controlled thermal-exposure study with the same cement paste mixture identified a bond reduction under sustained 200 °C [26]. In the present comparison, the mean difference of 9% is smaller than this reduction of 29%, which lies within the confidence interval of the difference (Section 3.1).

### 3.4. Limitations of the Push-Through Configuration

Because of the compression loading and the steel confinement ring (Section 2.4), the measured bond capacities refer to a confined bar under compression. The displacement at the free bar end excludes the elastic shortening of the bar and the joints, but a different joint stiffness may still change the distribution of slip along the bond length. Since the segments were butt-joined and the joints were loaded in compression, the cut faces were pressed against each other, so that the joined bars are expected to behave approximately like the continuous bar of a RILEM RC6 test in this respect. The results therefore characterise the bond of the joined bars under short-term compressive loading.

### 3.5. Transfer to WAAM

The present procedure resembles stud welding rather than genuine WAAM. Short segments of 10 mm (final segment 20 mm) were joined in single welding operations, which were separated by several printed layers. A similar stud-welding-like approach is used for segmented reinforcement in the extrusion-based AMoRC process [10,11]. Over the bond length of 80 mm, the present bars contain seven joints, each made once per joining cycle at intervals of 15.4 to 17.6 min. A WAAM-deposited bar with a mean layer height of 1.25 mm [24] consists of 64 layers over the same length, each a weld bead and a thermal cycle, deposited at much shorter intervals. In genuine WAAM, heat therefore accumulates over successive layers [16], and the overlapping thermal cycles in a given bar cross-section can produce higher sustained temperatures at the steel–concrete interface than in the present procedure, of the kind shown to degrade bond under controlled heating [26] and under cyclic thermal loading [27]. The present results thus represent the low-exposure end of the range between single joining operations and continuous WAAM deposition. For full WAAM integration, active cooling strategies [17,18] or a lower heat input per weld are therefore necessary. Drawn-arc stud welding, which completes a joint in a sub-second welding cycle [45], is one option for automating the segment-joining route with a defined heat input.

A further transfer aspect concerns joint cleanliness. In the present study, the joint faces were cleaned before each welding cycle, so that contamination by particles or cement paste was largely excluded. Moisture is a known source of diffusible hydrogen in arc welds, shown for example for moist stick electrode coatings [46], and contamination of the weld has been shown to change its microstructure and reduce its strength, for example in friction stir spot welds of steel [47]. In an automated SPI-WAAM process without intermediate cleaning, this adds to the qualification requirements for the welded joints, independently of the concrete-side bond behaviour measured here.

The heat input per joint depended on the manual procedure, since arc duration and filler addition varied from joint to joint (Section 2.2). Because the thermal exposure is the variable of interest, this variability of the manual procedure limits the present study. The thermography documents the resulting exposure of the bed surface (Section 3.2).

## 4. Conclusions

This study investigated the bond behaviour between reinforcement and concrete fabricated by Selective Paste Intrusion (SPI) under process-integrated thermal exposure from welding of steel segments, with adhesively bonded segments without heat input as the reference. Reinforcing bars with a diameter of 16 mm embedded vertically in the particle bed were tested in push-through (eight specimens per group from two production series). Under short-term compressive push-through loading, the welded bars reached a mean bond capacity close to that of the adhesively bonded reference, for the stud-welding-like scenario investigated here with seven joints per bar at 10 mm spacing and approximate bar-surface temperatures of at least 214 to 277 °C immediately after welding and 125 to 162 °C after 2 to 3 min. The results therefore indicate that joining reinforcement segments by stud-welding-like processes during SPI printing is a feasible route with respect to the bond under short-term compressive loading. Longer segments, within the clearance below the print heads (Section 2.2), reduce the number of welding cycles per bond length. For reinforcement produced by genuine Wire Arc Additive Manufacturing (WAAM), further investigations are required, in particular regarding heat accumulation, and questions of joint cleanliness are still to be addressed separately as a process-engineering task.

The welded and the adhesively bonded groups reach comparable bond capacities (20.5 MPa and 22.4 MPa). At 1.9 MPa (9%), the difference is smaller than the standard deviation of either group, and the coefficients of variation of the two groups are 22% and 21%. No discernible difference was therefore detected in this sample, while the 95% confidence interval of the difference (−7.0 to 3.1 MPa) shows that a reduction of up to 31% cannot be excluded with eight specimens per group from two production series. The scatter exceeds the range reported for pull-out testing. Since it is of the same size in both groups, it more likely originates from the fabrication steps common to both groups (SPI intrusion, manual segmentation and joining, core drilling) than from the thermal exposure, which only the welded group received.

Bond stress values at very small slips (*s* ≤ 0.01 mm) approach the resolution limit of the displacement measurement and show no consistent difference between the groups. All specimens failed by bar push-through and none by splitting, and the slip at maximum bond stress is similar in both groups. Because the weld beads change the joint geometry, the thermal exposure cannot be fully separated from the joint geometry in this comparison (Section 3.3). For the joining of segments by welding during printing, this combined effect is the one that acts. The results characterise the steel–concrete bond under short-term compressive loading (Section 3.4).

## 5. Outlook

Three steps follow from the present results. First, automated joining with a defined heat input per weld, for example by stud welding, where the process variant (drawn-arc versus capacitor-discharge according to DIN EN ISO 14555 [48]) governs the actual heat input, would eliminate the variability of the manual procedure and allow the remaining scatter to be attributed to the process rather than to the preparation. Second, the welded joints are to be characterised for structural use, in tension according to DIN EN ISO 17660-1 [49], under fatigue loading, and with respect to durability. Measurements of the bead geometry, hardness profiles across the joints, and microstructural analyses of the interface zone would separate the joint-related mechanisms discussed in Section 3.3. Third, the step towards in situ WAAM deposition combined with SPI, with its higher welding frequency and cumulative thermal load, is still to be taken. The temperature thresholds established in the controlled study [26] and the temperature measurements and cooling strategies quantified in the concept study [24] define the design space for this step.

## Figures and Tables

**Table 1:** Bond stresses at the four characteristic slip levels and slip at maximum bond stress: group mean ± SD and coefficient of variation (CV) of the eight specimens per group, and difference welded minus adhesively bonded with its 95% confidence interval (CI, Welch’s *t*-test). Values at s=0.001 and 0.01 mm are close to the resolution of the displacement measurement. Individual values are given in Table A1.

	Welded		Adhesively Bonded		Difference
Characteristic	$\bar{x}\pm \mathrm{SD}$	CV	$\bar{x}\pm \mathrm{SD}$	CV	[95% CI]
τ(0.001) (MPa)	1.8±3.3	182%	2.9±3.1	110%	−1.0 [−4.5, +2.5]
τ(0.01) (MPa)	6.3±3.5	56%	6.6±2.7	42%	−0.2 [−3.7, +3.2]
τ(0.1) (MPa)	13.3±3.0	23%	15.1±2.8	19%	−1.8 [−5.0, +1.3]
$\tau_{\max}$ (MPa)	20.5±4.6	22%	22.4±4.8	21%	−1.9 [−7.0, +3.1]
$s(\tau_{\max})$ (mm)	0.81±0.19	23%	0.69±0.18	26%	+0.12 [−0.07, +0.32]

## Data Availability

The data supporting the results of this study are available on Zenodo at https://doi.org/10.5281/zenodo.22050194.

## Appendix A

**Table A1:** Individual bond stresses (MPa) and slip at maximum bond stress (mm). Specimen designation: position P01 to P04 on the welded rail and P05 to P08 on the adhesively bonded rail (Figure 3c), followed by the production series (.1 or .2). Group means ($\bar{x}\pm \mathrm{SD}$) of the eight specimens per group are given.

Group	Specimen	τ(0.001)	τ(0.01)	τ(0.1)	$\tau_{\max}$	$s(\tau_{\max})$
Welded	P01.1	0.40	7.30	14.22	22.63	0.69
	P02.1	0.04	2.73	13.67	20.51	1.17
	P03.1	9.90	11.58	16.53	24.13	0.76
	P04.1	1.01	5.42	12.77	22.15	0.95
	P01.2	0.40	3.58	11.87	16.55	0.65
	P02.2	0.52	2.17	6.87	11.66	0.59
	P03.2	0.06	7.14	13.77	19.90	0.81
	P04.2	2.39	10.80	16.32	26.15	0.89
	$\bar{x}\pm \mathrm{SD}$	1.8±3.3	6.3±3.5	13.3±3.0	20.5±4.6	0.81±0.19
Adhesively bonded	P05.1	0.19	1.03	18.48	30.25	1.07
	P06.1	7.57	10.10	16.29	23.74	0.65
	P07.1	3.03	8.49	17.88	24.62	0.50
	P08.1	0.39	6.32	17.58	26.03	0.62
	P05.2	7.42	7.90	11.07	14.78	0.60
	P06.2	0.53	5.11	12.79	20.59	0.78
	P07.2	3.50	7.80	12.52	20.29	0.72
	P08.2	0.18	5.86	14.11	18.85	0.56
	$\bar{x}\pm \mathrm{SD}$	2.9±3.1	6.6±2.7	15.1±2.8	22.4±4.8	0.69±0.18
